# Quality of a Supporting Mobile App for Rheumatic Patients: Patient-Based Assessment Using the User Version of the Mobile Application Scale (uMARS)

**DOI:** 10.3389/fmed.2021.715345

**Published:** 2021-07-22

**Authors:** Antonia Lambrecht, Nicolas Vuillerme, Christina Raab, David Simon, Eva-Maria Messner, Melanie Hagen, Sara Bayat, Arnd Kleyer, Timothée Aubourg, Georg Schett, Axel Hueber, Johannes Knitza

**Affiliations:** ^1^Department of Internal Medicine 3 - Rheumatology and Immunology, Friedrich-Alexander University Erlangen-Nürnberg and Universitätsklinikum Erlangen, Erlangen, Germany; ^2^Université Grenoble Alpes, AGEIS, Grenoble, France; ^3^LabCom Telecom4Health, Univ. Grenoble Alpes & Orange Labs, Grenoble, France; ^4^Institut Universitaire de France, Paris, France; ^5^Deutsches Zentrum für Immuntherapie, Friedrich-Alexander-University Erlangen-Nürnberg and Universitätsklinikum Erlangen, Erlangen, Germany; ^6^Department of Clinical Psychology and Psychotherapy, Institute of Psychology and Education, University of Ulm, Ulm, Germany

**Keywords:** mobile applications (apps), mobile app rating scale, rheumatology, end-users, mobile health

## Abstract

**Introduction:** Mobile applications promise to improve current health care. However, current mobile app quality ratings are mostly physician-based. The aim of this study was (1) to assess the quality of the self-management app Rheuma Auszeit using the validated uMARS (User Version of the Mobile App Rating Scale) app quality assessment tool and (2) to evaluate the association between uMARS scores and patients' characteristics.

**Materials and Methods:** Consecutive patients with rheumatoid arthritis, psoriatic arthritis and spondyloarthritis were seen at the rheumatology clinic at university hospital Erlangen, Germany. They were asked to test Rheuma Auszeit, evaluate its quality using uMARS and complete a paper-based survey evaluating the individual preferences, attitudes and ehealth literacy. The association between uMARS scores and patients' characteristics was further explored.

**Results:** Between December 2018 and January 2019, a total of 126 patients evaluated Rheuma Auszeit using uMARS and filled out the paper-based survey. The median uMARS score was 3.9, IQR 0.7. Functionality was the domain with the highest rating (median 4.8, IQR 0.8), followed by aesthetics (median 4.0, IQR 0.7), information (median 3.5, IQR 0.8), and engagement (median 3.2, IQR 1.0). Subjective quality was average (median 3.0, IQR 1.0). The lowest scoring individual item was customization with a median of 2.5/5. Lower functionality scores were reported among older female rheumatic patients (*P* < 0.004). Older male rheumatic patients reported a higher subjective quality score (*P* < 0.024). Perceived disease activity and disease duration did not significantly correlate with any uMARS subdomain scores. eHealth literacy significantly correlated with functionality uMARS subdomain ratings (Rho = 0.18; *P* < 0.042). Preferred time of app usage significantly correlated with engagement (Rho = 0.20; *P* < 0.024), functionality (Rho = 0.19; *P* < 0.029), total uMARS score (Rho = 0.21; *P* < 0.017) and subjective quality score (Rho = 0.21; *P* < 0.017). The vast majority of rheumatic patients would consider recommending Rheuma Auszeit to other patients (117/126; 92.9%).

**Conclusion:** Rheuma Auszeit was well-accepted by German patients suffering from rheumatoid arthritis, psoriatic arthritis and ankylosing spondyloarthritis. Lacking customization could lead to low app compliance and should be improved. Lower functionality scores among older female rheumatic patients highlight the need for patient education. The study underlines the potential and feasibility of therapeutic complementary digital solutions in rheumatology.

## Introduction

Rheumatology encompasses a variety of chronic inflammatory diseases that require immunosuppressive treatment. Despite more effective medical treatment becoming available to patients, many patients are unable to reach clinical remission. An integrative, holistic treatment approach might improve patients' quality of life. Due to the wide adoption of smartphones (1), medical apps could become an effective tool (2–5) to promote predictive, preventive, personalized and participatory (“P4”) medicine (6).

The European Alliance of Associations for Rheumatology (EULAR) anticipated the considerable potential of mobile apps to aid rheumatic patient disease self-management and recently published guidelines (3) to support the implementation into clinical care. Medical apps are already being used in clinical routine by an increasing number of rheumatologists (7). However, little knowledge of the quality of existing apps (8) and evidence (9) supporting their effectiveness and acceptance currently prevents widely adoption (10). In a recent physician-based AppStore analysis (4), Rheuma Auszeit received the highest rating among sixteen identified apps meeting inclusion criteria. Notably, Rheuma Auszeit was the only app, mainly developed by rheumatic patients (German League against Rheumatism). Most of the apps provided symptom tracking, whereas Rheuma Auszeit delivers video and audio instructions for mental and physical exercises.

Mobile apps created for patient use are currently primarily assessed exclusively by physicians (4, 5), yet it has been repeatedly stressed to include end-user (patient) evaluations (1, 4, 11–13). The perceived app quality is subjective and could significantly differ between patients and physicians, similar to perceived disease activity (14). To enable a user-centric app evaluation, the user version of the Mobile App Rating Scale (uMARS) has been created (15). It assesses the dimensions of engagement, functionality, aesthetics, information, and subjective quality on 5-point scales and has been used to evaluate the usability of dengue fever apps (16), mental health apps (17) and wearable sensor-based biofeedback systems (18). User-centered app evaluations are crucial to improve app design, effectiveness and importantly app adherence (4). In order to maximize app effectiveness and long-term usage, continuous improvement and personalization are necessary (1, 4, 9, 12, 13).

The aim of this study was to assess the quality of Rheuma Auszeit by rheumatic patients using uMARS and to explore associations between uMARS scores and patient characteristics.

## Materials and Methods

Consecutive patients with rheumatoid arthritis (RA), psoriatic arthritis (PsA) and spondyloarthritis (SpA) were recruited between December 2018 and January 2019 at the rheumatology clinic at University hospital Erlangen, Germany. The study was approved by the local Ethics committee (No. 418-18B) and conducted referring to good clinical practice. All patients provided informed consent and were then asked to (1) evaluate the app Rheuma Auszeit using uMARS and to (2) complete a paper-based survey evaluating the individual preferences, attitudes and eHealth literacy. The association between uMARS scores and patient characteristics were further explored.

### Rheuma Auszeit

**Figure 1:**
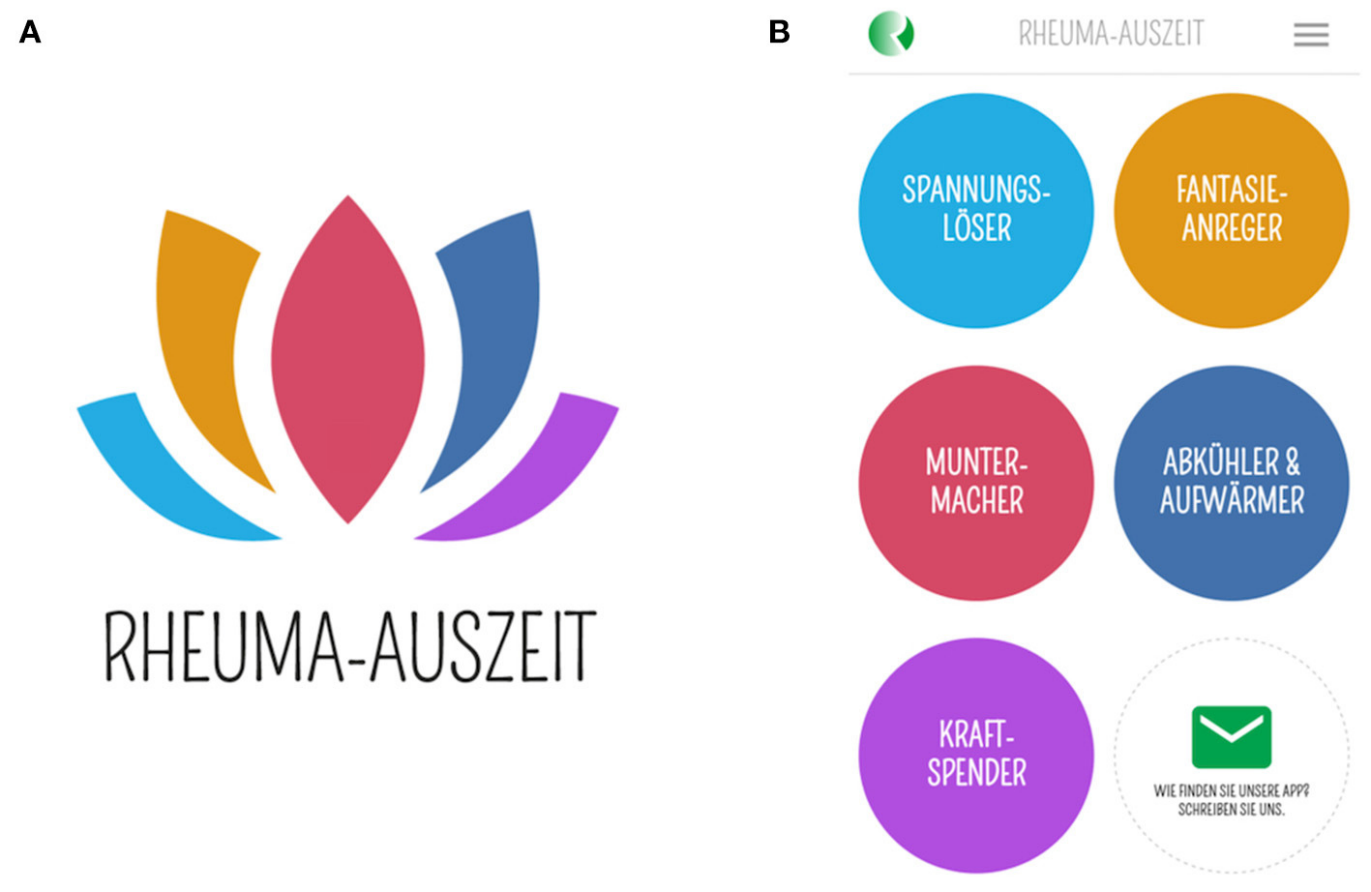
Screenshots of the Rheuma Auszeit app: **(A)** logo, **(B)** main menu including the following options: stress relief, imagination stimulator, pick-me-up, cool down & warm up, power donor, feedback. Images provided by the developer, German League against Rheumatism.

Rheuma Auszeit (Figure 1) has been developed by the German RMD patient organization Rheuma-Liga and is available to download for free in the German App Store and Google Play store. It offers rheumatic patients, irrespective of the exact rheumatic disease, practical self-management recommendations that can immediately be applied to support patients coping with rheumatic pain. The app consists of audio, images and videos that should guide patients. Exercises manuals include muscle relaxation, self-massage, mind journeys, movement training and cold/hot treatments. There is no predetermined order of exercises and patients are free to select their preferred exercises. Importantly, symptom tracking/monitoring offered by various other apps (4) is not offered by this app. All material can be downloaded to support offline usage.

### App Evaluation Using uMARS

Local study personnel explained the goal and functions of the app and gave a short demonstration. As recommended by the uMARS developers (15), patients tested Rheuma Auszeit for at least 10 min using tablets (IPads). Patients were then asked to evaluate the app quality employing the paper version of the German version of the uMARS (15), which is a simplified user version of the MARS (19, 20) including 20 items instead of the original 23. MARS has been used to evaluate the quality of a multitude of apps and its construct validity, reliability and objectivity has been recently validated in a multicenter study (19). Both rating scales assess the five dimensions: engagement, functionality, aesthetics, information, and subjective quality on 5-point scales. Instead of the original 7 information quality MARS items, uMARS consists of four information quality items: quality of information, quantity of information, visual information, credibility of source. The subjective quality dimension is based on four questions also using 5-point scales: willingness to recommend the app, anticipated app usage frequency, willingness to pay for the app, and overall rating.

### Patient Survey

Besides the app evaluation, patients were asked to complete a paper-based survey regarding individual sociodemographic details, mhealth preferences, attitudes and eHealth literacy. Patients' eHealth literacy was measured using the validated German version (21) of the eHealth Literacy Scale (eHEALS) (22). It is based on a 5-point Likert scale and includes eight statements concerning self-perceived eHealth literacy. We previously published exact survey details and results from a larger patient collective (1). In this study survey data was only used from patients that evaluated Rheuma Auszeit.

### Statistical Analysis

The statistical analysis was split into three distinct steps:

- Patient characteristics were summarized using medians, interquartile ranges, means, standard deviations, counts, and percentages as appropriate. Descriptive statistics were computed for describing distributions of variables related to uMars score and subjective quality among patients. In details, boxplots and distribution histograms were used to summarize scores distributions from the whole population regarding engagement, functionality, aesthetics, information, total score and subjective quality variables. Among these variables, (1) engagement, (2) functionality, (3) aesthetics and (4) information variables resulted from a combination of several other variables averaged together, respectively (1) entertainment, interest, customization, interactivity, target group, (2) performance, ease of use, navigation, gestural design (3) layout, graphics, visual appeal, (4) quality of information, quantity of information, visual information and credibility of source. Thus, supplementary boxplots were computed for summarizing the distribution of each of these combination variables. Finally, boxplots computed from the whole population for describing the main variables, i.e., engagement, functionality, aesthetics, information, total score and subjective quality, were visually compared with subgroups of the patient populations related to gender and disease. As such, violin plots were used to compare the whole patient population with (1) axial spondyloarthrisis, (2) psoriatic arthritis, (3) rheumatoid arthritis. Similarly, violin plots were also used to compare the whole patient population with (1) female and (2) male patients.
- Association between main variables of interest were calculated by means of Spearman's correlation test. Spearman's rho and its corresponding *p*-value were calculated for correlating each mobile app rating scales (i.e., engagement, functionality, aesthetics, information, total score and subjective quality variables) with variables related to (1) sociodemographics (population age, female age, male age), (2) health characteristics (disease duration, disease activity), (3) mHealth characteristics (ehealth literacy, prefered time for app usage) and (4) the importance of app characteristics (interactivity, design, usability, data security). Correlations were calculated from the whole patient population and for each disease subgroup, separately.
- Each of the variables described above were compared according to (1) gender (female vs. male patients) and to disease (axial spondyloarthritis vs. psoriatic arthritis vs. rheumatoid arthritis patients) using Mann-whitney comparative tests. Total score and Subjective quality variables were compared for (1) the whole population, (2) female, (3) male, (4) axial spondyloarthritis, (5) psoriatic arthritis, and (6) rheumatoid arthritis patients, separately.

The level of significance was set as *p* < 0.05 in all statistical tests. All statistical calculations were completed using the R software environment (version 3.1.0; R Foundation for Statistical Computing, Vienna, Austria).

## Results

### Patient Characteristics

Patient demographic information is displayed in Table 1. In total, 126 rheumatic patients, of which 76 (60.3%) were female, completed the study. This population consisted of 59 (46.8%) RA, 27 (21.4%) SpA, and 40 (31.7%) PsA patients. Mean disease duration was 7.8 years (SD 7.2) and mean patient global disease activity was 4.1 (SD 2.4).

**Table 1:** Demographic and health characteristics.

**Characteristic**	**Values (*N* = 126)**
Age (years), mean (SD)	52 (14.1)
**Age (years)**, ***n*** **(%)**	
18–39	34 (27.0)
40–59	48 (38.1)
≥60	44 (34.9)
**Gender**, ***n*** **(%)**	
Female	76 (60.3)
Male	50 (39.7)
**Diagnosis**, ***n*** **(%)**	
Rheumatoid arthritis	59 (46.8)
Axial spondyloarthritis	27 (21.4)
Psoriatic arthritis	40 (31.7)
Patient global assessment of disease activity (0–10), mean (SD)	4.1 (2.4)
Disease duration (years), mean (SD)	7.8 (7.2)
**Disease duration (years)**, ***n*** **(%)**	
≤ 1	27 (21.4)
2–5	32 (25.4)
>5	67 (53.2)

Table 2 displays the patient mHealth attitude and current usage. The majority of patients used smartphones on a regular basis, almost half of the patients regulary used social media and only a minority regularly used activity trackers. Mean eHealth literacy using eHEALS was (26.1/40; SD 7.1). Most patients believed that medical apps are helpful for their health (89/126; 70.6%) and were willing to use them in the future (83/126; 65.9%), preferably for a maximum of 5-15 min (43/126; 34.1%). Nearly all patients (125/126; 99.2%) were willing to share app data for research purposes. The majority of patients 86/126; 68.3%) was previously looking for treatment options on the Internet and was interested in an app including physical exercises and stress reduction activities (94/126; 74.6%). Most patients wanted official app recommendations from the national society of rheumatology (98/126; 77.8%) and only a small minority was aware of useful rheumatology apps/websites (10/126; 7.9%) or previously participated in an online health program (2/126; 1.6%). Usability and data security were rated as more essential app features (10) than design (5) and interactivity (5) by patients.

**Table 2:** Patient mHealth attitude and usage.

**Characteristic**	**Values (*N* = 126)**
**Patients regularly using**, ***n*** **(%)**	
Smartphone	115 (91.3)
Tablet	55 (43.7)
Activity tracker	10 (7.9)
Social media	61 (48.4)
eHealth literacy (8–40), mean (SD)	26.1 (7.1)
Patients believing medical apps are helpful for their health, *n* (%)	89 (70.6)
Patients willing to transfer app data for research purposes, *n* (%)	125 (99.2)
Patients willing to transfer data to physician with app, *n* (%)	70 (55.6)
Patients aware of useful rheumatology websites or apps, *n* (%)	10 (7.9)
Patients that participated in an online health programm, *n* (%)	2 (1.6)
Patients looking for treatment options on the Internet, *n* (%)	86 (68.3)
Patients interested in apps with online physical exercises and stress reduction activities, *n* (%)	94 (74.6)
Patients that want official app recommendations from national society of rheumatology, *n* (%)	98 (77.8)
Patients willing to use medical apps, *n* (%)	83 (65.9)
**Preferred time of medical app usage (minutes)**, ***n*** **(%)**	
Not at all	43 (34.1)
0–5	22 (17.5)
5–15	43 (34.1)
15–30	17 (13.5)
>30	1 (0.8)
**Importance of app characteristics (0–10), median**	
Interactivity	5
Design	5
Usability	10
Data security	10

### Rheuma Auszeit uMARS Rating

**Figure 2:**
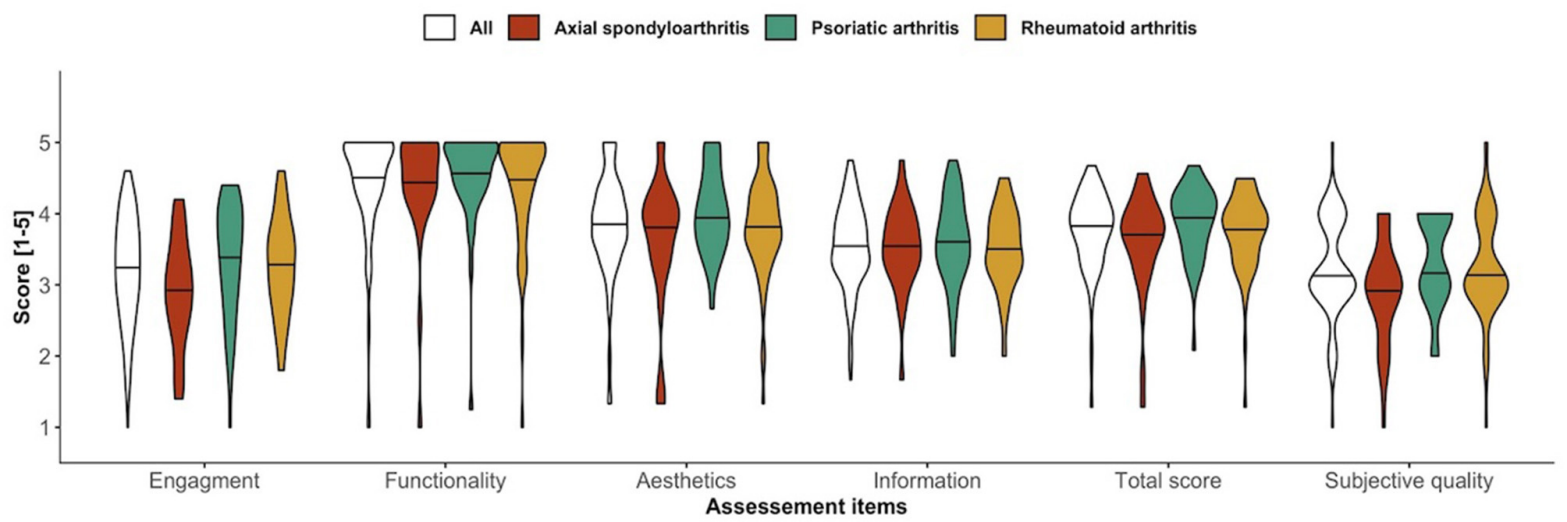
Violin plots of uMARS subdomains, total score and subjective quality according to disease group.

Figure 2 shows the uMARS subdomain ratings, total score and subjective quality according to disease group. The overall median uMARS score was 3.9/5, IQR 0.7. Functionality was the domain with the highest rating (median 4.8, IQR 0.8), followed by aesthetics (median 4.0, IQR 0.7, information (median 3.5, IQR 0.8), and engagement (median 3.2, IQR 1.0). Engagement scores were significantly lower for SpA patients (*P* < 0.047) and PsA patients (*P* < 0.016) compared to RA. Subjective quality was average (median 3.0, IQR 1.0) and was significantly lower in SpA compared to PsA patients (*P* < 0.013).

**Figure 3:**
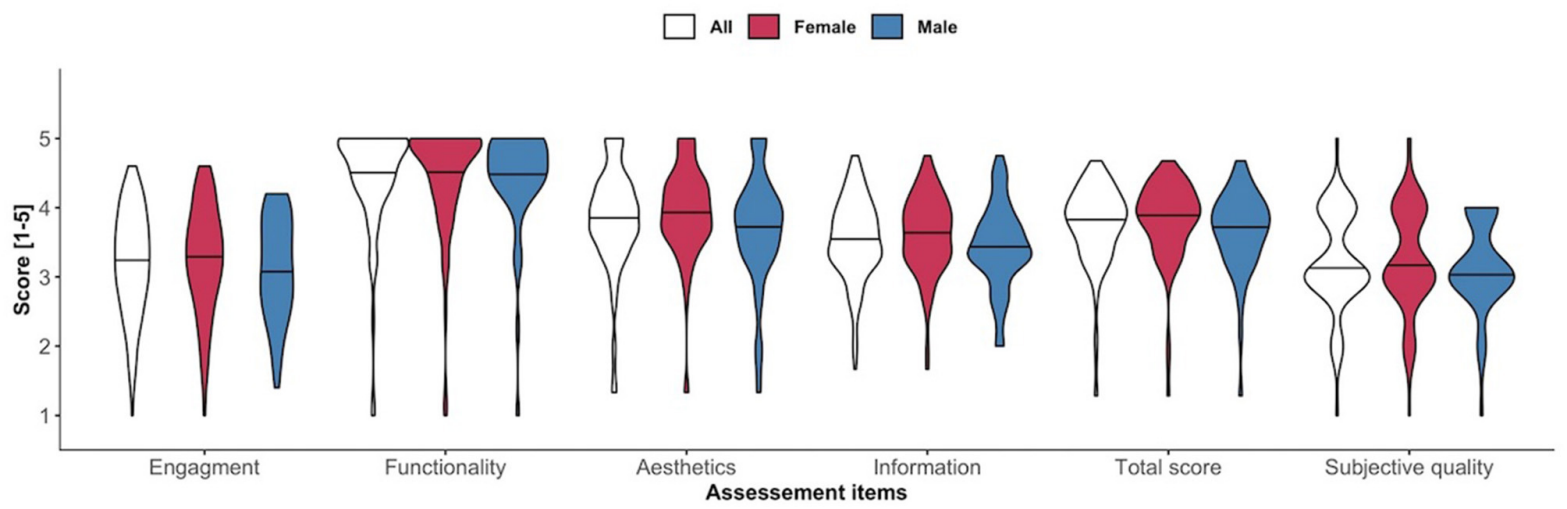
Violin plots of uMARS subdomains, total score and subjective quality according to sex.

**Figure 4:**
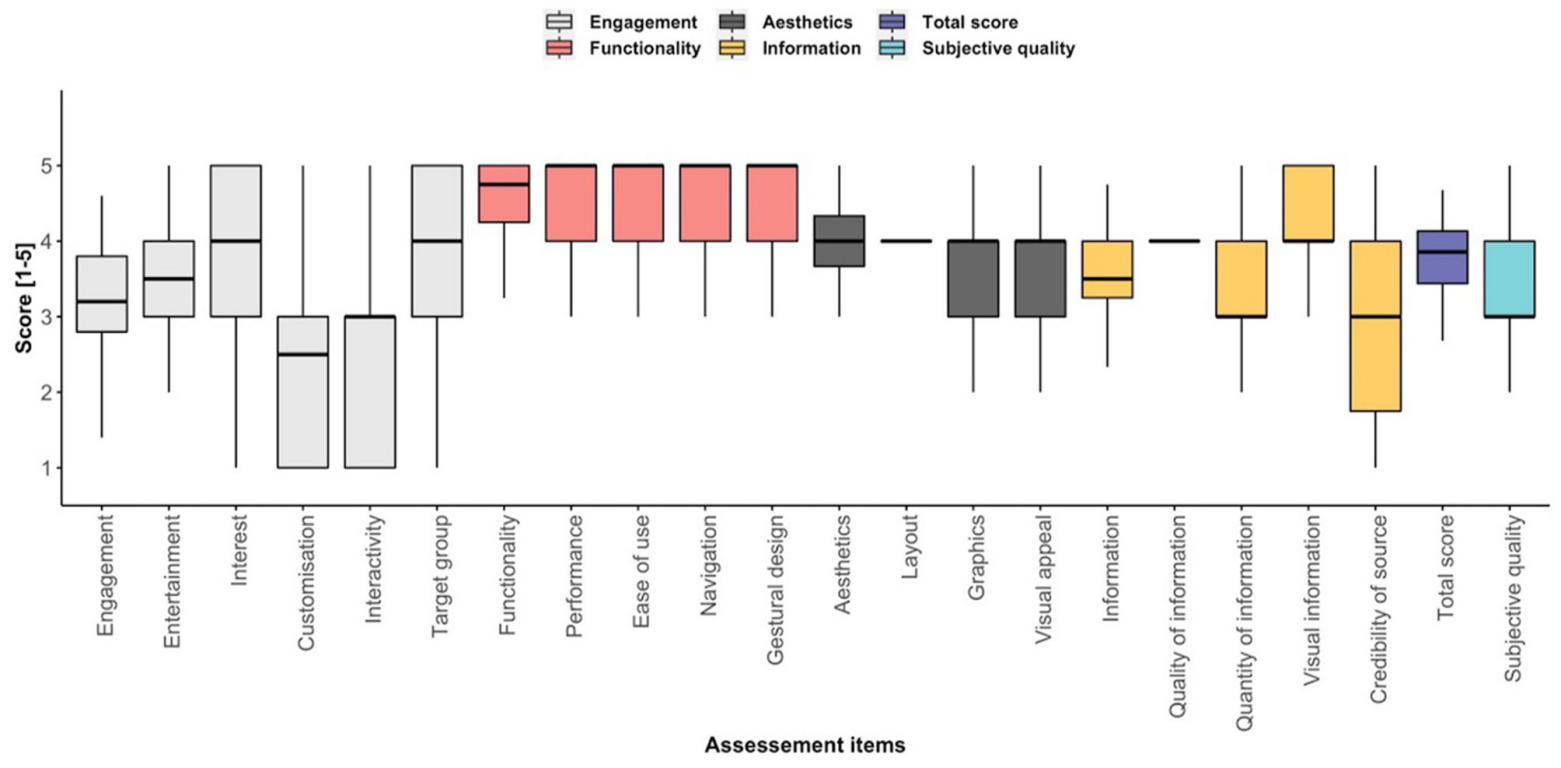
Box plots of single uMARS subdomain items, total score and subjective quality.

Figure 3 shows no significant gender-based differences regarding uMARS subdomain ratings, total score and subjective quality. Figure 4 presents the single uMARS items of the four subdomains, uMARS total score and subjective quality. The highest individual item scores were the functionality subdomain items (performance, ease of use, navigation and gestural design), each with a median of 5/5. The lowest scoring item was customization with a median of 2.5/5.

Table 3 lists the subjective app quality uMARS item ratings. Only a minority of patients would not at all recommend Rheuma Auszeit (9/126; 7.1%) and most patients (61/126; 48.4%) would maybe recommend it. Similarly, only 14/126 (11.1%) patients think that they would never use Rheuma Auszeit. 10/126 (7.9%) patients would definitely pay for this app. The majority of patients assigned an average rating (68/126; 54.0%).

**Table 3:** Subjective app quality uMARS item ratings.

**Subjective app quality uMARS items**	**Values (*N* = 126)**
**Would you recommend this app to people who might benefit from it?** ***n*** **(%)**	
1. Not at all	9 (7.1)
2.	12 (9.5)
3. Maybe	61 (48.4)
4.	27 (21.4)
5. Definitely	17 (13.5)
**How many times do you think you would use this app in the next 12 months if it was relevant to you?** ***n*** **(%)**	
1. None	14 (11.1)
2. 1-2	11 (8.7)
3. 3–10	43 (34.1)
4. 10–50	48 (38.1)
5. >50	10 (7.9)
**Would you pay for this app?** ***n*** **(%)**	
1. Definitely not	63 (50.0)
2.	0 (0.0)
3.	53 (42.1)
4.	0 (0.0)
5. Definitely yes	10 (7.9)
**What is your overall (star) rating of the app?** ***n*** **(%)**	
1. One of the worst apps I've used	2 (1.6)
2.	13 (10.3)
3. Average	68 (54.0)
4.	41 (32.5)
5. One of the best apps I've used	2 (1.6)

Supplementary Table 1 displays the correlations between uMARS ratings, users' socio-demographics, health characteristics and eHealth literacy. We observed a significant correlation with increasing age and lower functionality uMARS subdomain ratings for female patients (Rho = −0.33; *P* < 0.004). Increasing age correlated positively with subjective quality (Rho = 0.32; *P* < 0.024) and total uMARS score (Rho = 0.25; *P* < 0.083) for male patients.

Perceived disease activity and disease duration did not significantly correlate with any uMARS items. eHealth literacy significantly correlated with functionality uMARS subdomain ratings (Rho = 0.18; *P* < 0.042). Preferred time of app usage significantly correlated with engagement (Rho = 0.20; *P* < 0.024), functionality (Rho = 0.19; *P* < 0.029), total uMARS score (Rho = 0.21; *P* < 0.017) and subjective quality score (Rho = 0.21; *P* < 0.017). Importance of usability correlated with functionality scores (Rho = 0.18; *P* < 0.048) and total scores (Rho = 0.19; *P* < 0.035).

## Discussion

In a previous German app store analysis (4), Rheuma Auszeit received the highest physician-based MARS rating (4.2/5). It was however unclear if rheumatic patients share this positive perception. This study now complements the previous evaluation by presenting a detailed patient perspective, as demanded in the published EULAR recommendations (3). To our knowledge, Rheuma Auszeit is the first German rheumatology app that has been tested by both, physicians and patients. Our study provides valuable patient insights regarding the app's usability in terms of its functions, engagement, design, information, and subjective qualities.

This study shows that Rheuma Auszeit is well-accepted among rheumatic patients. In particular, the app received high functionality and aesthetics ratings. Most patients stated that they would use the app 3–50 times (91; 72.2%) during the next 12 months and only a minority of patients (14/126; 11.1%) would not use the app at all. We did not observe significant correlations between disease activity, disease duration and total uMARS score.

The overall uMARS score was 3.9/5 compared to the physician-based MARS score (4.2/5) (4). Similar to the previous physician-based MARS analysis (4), subjective quality was significantly (*P* < 0.0001) lower compared to the overall uMARS score. The lower subjective ratings could be due to a general German conservativeness. Importantly our patient results are in line with physician evaluations rating the engagement subdomain as the worst uMARS subdomain (4) and showing no significant gender rating differences. We believe that different disease specific app expectations could have caused the differences regarding the uMARS engagement subsection. For example SpA patients are likely to be more engaged if spine associated features are addressed in the app.

Patients were reluctant to pay for the app and actively recommend it to other patients. Most patients would welcome such app recommendations from the national society of rheumatology. The German digital healthcare act (Digital Versorgung Gesetz-DVG) allows to partially overcome these implementation barriers, as physicians can now prescribe designated medical apps to patients, which are then reimbursed by insurance companies.

Rheuma Auszeit (translated: Rheuma Timeout) is unique, as it was the only app in a recent review mainly developed by rheumatic patients offering coping techniques (4). The top two app features rheumatic patients named in a previous survey were information (medication and disease) and exercises to reduce stress and pain (1). Rheuma Auszeit addresses the latter. Most other previously identified German rheumatic apps focus on symptom tracking (4), however offer no techniques to reduce stress and pain. Lalloo et al. showed that symptom tracking alone can lead to a clinically meaningful reduction in pain intensity in a population with juvenile idiopathic arthritis (23). On the other hand, Seppen et al. showed that pain positively mediated app usage in RA patients but some patients identified symptom tracking (reminding patients of their disease) as a barrier for app usage (24). This suggests that symptom tracking frequency should be discussed with patients via shared-decision-making (SDM). Shaw et al. recently showed that patient-reported outcome results should be discussed during face-to-face time to enhance the patient-provider interaction (25).

We believe that rheumatic patients need to receive proper instructions on how to use medical apps similar to other medical products. This personalized training will likely improve app adherence and effectiveness, however will most likely not be carried out by the treating physician due to the pressing time constraints. The lack of information on available tools and supporting evidence are main barriers that delay wide adoption. Our study showed lower functionality scores among older female patients (*P* < 0.004) and higher subjective quality scores among older male patients (*P* < 0.024). This suggests that not all rheumatic patients might benefit equally from mHealth solutions and instructions need to be personalized as well.

The high overall uMARS score and number of rheumatic patients regularly using smartphones imply that a longitudinal clinical study is feasible. The low customization score suggests however that such a trial will likely result in low app adherence, especially since app retention in general is an imminent mHealth barrier (26) also present in rheumatology (24, 27). In our opinion, customizability should be improved by gathering additional qualitative feedback from rheumatic patients before initiating a longitudinal clinical study evaluating clinical benefits of the app.

This study has some limitations. The analysis was solely based on quantitative feedback from a single evaluation tool based on short app usage. No sample size calculation was carried out. Qualitative feedback, similar to Herbuela et al. (16), would have added valuable depth and details to our analysis and are planned to be carried out. Furthermore, the disease selection limits the generalizability to other rheumatic diseases and only patient-reported items were collected in the study. We received a lot of positive feedback especially from older patients that would not have come in contact with Rheuma Auszeit otherwise. This feedback was however not properly measured. Furthermore, Rheuma Auszeit is currently only available in German, limiting the potential international impact. The German uMARS version which we used has not been published yet, however the items are identical to the published German MARS translation (28). The System Usability Scale (SUS) (24, 29) and Net Promoter Score (NPS) (24) are increasingly being used in app evaluation studies and could have enhanced our work.

## Conclusion

Rheuma Auszeit is well-accepted among patients suffering from different rheumatic diseases, different ages and digital competencies. Physicians and other health care professional should be encouraged to integrate mHealth into their clinical routine. Lacking customizability of Rheuma Auszeit diminished the total uMARS score and will likely limit long-term app usage by patients.

## Data Availability Statement

The raw data supporting the conclusions of this article will be made available by the authors, without undue reservation.

## Ethics Statement

The studies involving human participants were reviewed and approved by ethics committee of the Friedrich-Alexander University Erlangen-Nürnberg (FAU); (No. 418-18B). The patients/participants provided their written informed consent to participate in this study.

## Author Contributions

AL and JK wrote the draft manuscript. AL, JK, TA, and NV performed the statistical analysis. All authors reviewed the draft and provided comments for changes, approved the final manuscript.

## Conflict of Interest

The authors declare that the research was conducted in the absence of any commercial or financial relationships that could be construed as a potential conflict of interest.
