# Supplementary figures and images for: Quality of a Supporting Mobile App for Rheumatic Patients: Patient-Based Assessment Using the User Version of the Mobile Application Scale (uMARS)
Source: Front Med (Lausanne). 2021 Jul 22;8:715345. doi: 10.3389/fmed.2021.715345 (PMC8339429; doi:10.3389/fmed.2021.715345)

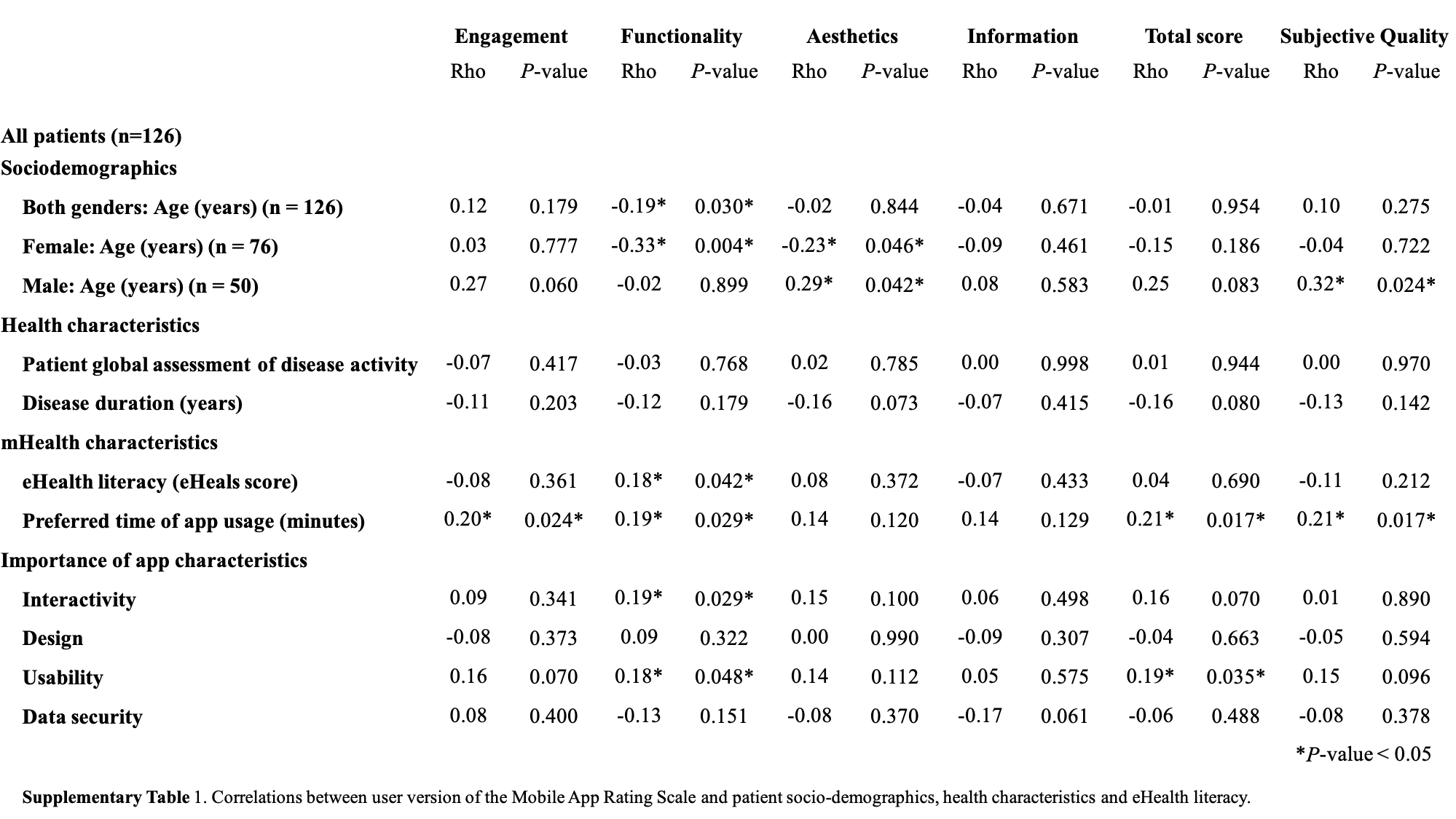

Supplement: Supplementary file 1 [file Image_1.PNG]
